# An Improved Modified Universal Ultra-Wideband Antenna Designed for Step Frequency Continuous Wave Ground Penetrating Radar System

**DOI:** 10.3390/s19051045

**Published:** 2019-03-01

**Authors:** Yuxuan Wu, Feng Shen, Yue Yuan, Dingjie Xu

**Affiliations:** School of Instrumental Science and Engineering, Harbin Institute of Technology, No. 92, Xidazhi Street, Nangang District, Harbin 150001, China; 18S001037@stu.hit.edu.cn (Y.W.); yyuansept@163.com (Y.Y.); xdj1966@hit.edu.cn (D.X.)

**Keywords:** antenna, ground penetrating radar, ultra-wideband, optimization method, Step Frequency Continuous Wave

## Abstract

Step Frequency Continuous Wave Ground Penetrating Radar (SFCW-GPR), as a tool for nondestructive testing of shallow soil surface targets, the realization of the function of SFCW-GPR is mainly based on the theory of refraction, reflection and scattering of electromagnetic wave in the discontinuity of dielectric constant. So, the UWB antenna system, an important part of SFCW-GPR, becomes more indispensable. In this paper, an improved modified universal antenna is designed, simulated and fabricated. Based on a typical Bow-tie antenna, it is modified by the methods of lumped loads, cavity-backed loading and structure loading. The simulated and measured results show that the UWB antenna has 1.36 GHz bandwidth from 0.64 to 2.0 GHz with three resonant wavelength peaks, and having been modified and improved, the UWB antenna performances including voltage standing-wave ratio (VSWR), input impedance, the boresight gain and current distribution, are much better than the typical Bow-tie antenna. In addition, the results of verification experiment of Step Frequency Continuous Wave (SFCW) show that the antenna can be applied to the working scenarios of SFCW-GPR.

## 1. Introduction

Ground Penetrating Radar (GPR) is an effective tool to identify and locate an underground target less than 50 m from the surface by the fact of the reflection and scattering theory of electromagnetic wave in discontinuous medium, because of its advantages in the detection process including fast speed, continuous and high imaging resolution. GPR has been widely used in engineering, geological exploration and other fields recently [1,2]. Currently, the GPR system can be divided into three types: Pulse-GPR, Frequency Modulated Continuous Wave GPR (FMCW-GPR), Step Frequency Continuous Wave GPR (SFCW-GPR). Compared with the other two types, the Step Frequency signal transmitted by SFCW-GPR has many other advantages including high sensitivity, high precision and ease of processing and analysis.

Ultra-wideband (UWB) technology, which was used in the field of wireless communication early on, has now been applied to the field of GPR by many researchers because of its strong penetrability. Noted in Reference [3], in the most recent version of the FCC Part 15 rules, the demarcation between wideband and UWB was decreased to 0.20 (20%). However, noted in References [3,4], the OSD/DARPA Review Panel states that signals having fractional bandwidths greater than 0.25 (25%) are ultra-wideband (UWB) and this definition is selected in this paper. In these definitions, the FCC and OSD/DARPA classification schemes use the fractional bandwidth, *B_F_*,

$$B_{F}=2\frac{f_{h}-f_{l}}{f_{h}+f_{l}}$$

as a frequency-independent dimensionless quantity to categorize signals and systems. In this formula, *f_h_* is the highest frequency and *f_l_* is the lowest frequency. Based on the authors’ suggestion [3], the OSD/DARPA UWB is selected as the definition in this paper. This classification is shown Table 1. 

UWB antenna is one of the most critical components of SFCW-GPR system because it is used to transmit the UWB signal by SFCW-GPR system. However, in order to keep the antenna’s fractional bandwidth at no less than 25%, which is the lower limit of frequency of UWB antennas, the size of the antenna increases with the decline of antenna’s working frequency. Recently, UWB antennas are widely used for wireless communication, target detection, and wireless vital signs monitoring. For example, for wireless communication a quad-notched UWB antenna is presented, which can filter the lower wireless local area network (WLAN), upper WLAN, worldwide interoperability for microwave access (WiMAX), and the Indian national satellite (INSAT) frequency bands operating at 5.15–5.35 GHz, 5.72–5.8 GHz, 3.30–3.60 GHz, and 4.50–4.80 GHz [5]. For target detection, a UWB frequency scanning array antenna is presented [6]. For wireless vital signs monitoring, a resonator integrated UWB antenna is presented, which can achieve UWB and Single/Dual continuously tunable-notch behaviors [7].

Also, various UWB antennas, including Vivaldi antenna and the horn antenna, have been investigated for the GPR application in the past few years [8,9]. Such antennas demonstrated good performance in GPR application, but they are too large to be portable. On the other hand, the slot antenna is another kind of widely used antenna in GPR system with the good performance in time domain and frequency domain. However, it is difficult for a slot antenna to achieve low dispersion, which limits its application in impulsive GPR system [10]. The planar spiral antenna (PSA) is also a popular choice of UWB antenna and Archimedean spiral antenna (ASA) is one of basic PSAs [11,12]. The ASA exhibits many advantages, including wide impedance bandwidth, simple fabrication, but this type of antenna has relatively complex feeding network and unfixed phase center, which will result in serious distortion when transmitting and receiving wideband signals [13].

Compared with the above antennas, Bow-tie antennas with simple feeding network and good phase stability are also widely used in GPR systems owing to their low profile and small dispersion, but the gain of a Bow-tie antenna is usually less than 4 dBi, which limits its development [10]. So, there are many optimizations proposed to improve its electrical performance. A half-ellipse antenna (HEA), which is an improvement of the Bow-tie antenna, is presented [14]. The proposed HEA has a fractional bandwidth of 100%, which is also much wider than typical Bow-tie antennas. In addition, a reflector-backed folded planar Bow-tie antenna (RF-PBA) for GPR is presented to improve the gain of Bow-tie antenna and its final design ensures a fractional bandwidth of 100%, and a boresight gain between 5 and 12 dBi within the frequency band of interest [15].

**Figure 1:**
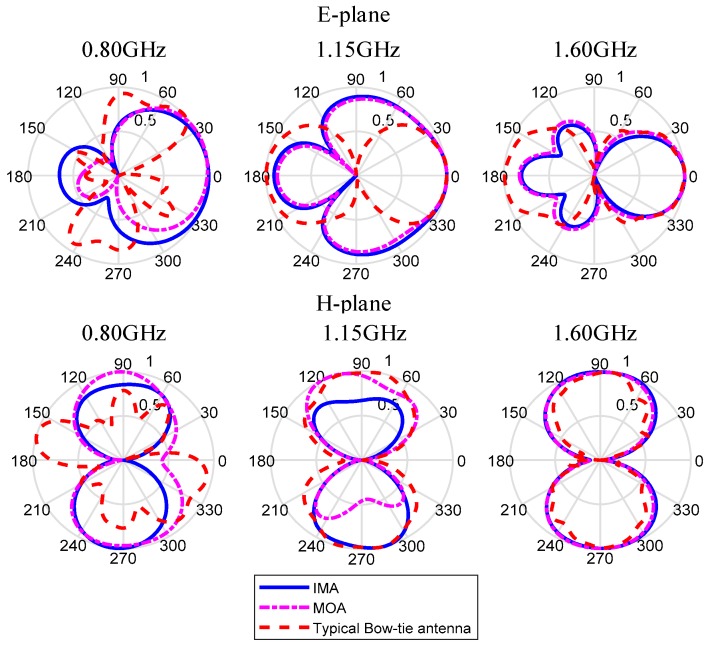
The E-plane and H-plane radiation pattern of typical Bow-tie antenna, MOA, and IMA.

In this paper, based on Bow-Tie antennas, an improved modified UWB antenna for SFCW-GPR is presented. In order to increase the antenna’s bandwidth on ultra high frequency (UHF), some improvements are made. Firstly, two angles in every triangle fin of the Bow-tie antenna are removed, because the size of the antenna can be reduced without its electrical performance changed. Secondly, a slot, which is used as capacitive load to improve the antenna input impedance, is etched on each side of triangle fins. Thirdly, there are two 50-ohm resistors placed at either terminal of each slot. Finally, the UWB antenna has operation bandwidth of 1.36 GHz from 0.64 to 2.0 GHz and its fractional bandwidth is over 100%. Compared with the typical Bow-tie antenna which operates within 0.7 to 0.9 GHz, the VSWR and input impedance of UWB antenna perform much better and it also has an evenly distributed current which helps to achieve a highly directive radiation pattern [16]. Besides, the peak boresight gain of designed antenna is higher than 5 dBi and the cavity is used to improve the directivity of antenna based on the image theory. Simulated and measured frequency domain results are presented to validate the use of the antenna in SFCW-GPR. The simulated performances of typical Bow-tie antenna, the modified antenna (MOA), and the improved antenna (IMA) are shown in Table 2 and Figure 1.

## 2. Materials and Methods

In this section, the materials and methods of antenna are introduced, and the whole design process can be divided in two steps: the first step is modification, in the step of which the structure of typical antenna is changed a lot; The next step is improvement, in the step of which the structure of the modified antenna is fine-tuned in order to improve the antenna’s electrical performance. In addition, the performances of the modified antenna (MOA) and the improved antenna (IMA) are simulated and compared below.

### 2.1. The Method of Modification

The major objective of this design is to detect the subsurface targets, most of which are located in several meters from the Earth’s surface. It is necessary for the GPR to equip a set of UWB antenna because of its SFCW signal system. In order to equip to the SFCW-GPR system with operation bandwidth of 1.36 GHz from 0.64 to 2.0 GHz, an antenna is characterized as follows:

- Has much larger impedance bandwidth than typical Bow-tie antenna;
- The VSWR in the whole working bandwidth is supposed to be less than 2.0 and exhibit high homogeneity;
- Has a boresight gain more than that of typical Bow-tie antenna;
- The real part of input impedance is around 50-ohms;
- The imaginary part of input impedance is around 0-ohms.

The prerequisite for microwave measurement is that the antenna must have relatively small size, a directive radiation pattern, high gain and wide fractional bandwidth. It is obvious that the Bow-tie antenna sensor can meet those requirements [14,17,18]. Based on the References [19,20], the Bow-tie antenna is a two-dimensional form of finite biconical antennas. Therefore, the input impedance of a Bow-tie antenna can be calculated by:

(1) $$Z_{in}=Z_{k}\frac{Z_{k}+jZ_{m}\tan\frac{2\pi R}{\lambda }}{Z_{m}+jZ_{k}\tan\frac{2\pi R}{\lambda }}$$

where *Z_k_* is a characteristic impedance of the antenna, *Z_in_* is an input impedance of the antenna, *Z_m_* is a load impedance, R is a side length of the triangle fin. 

**Figure 2:**
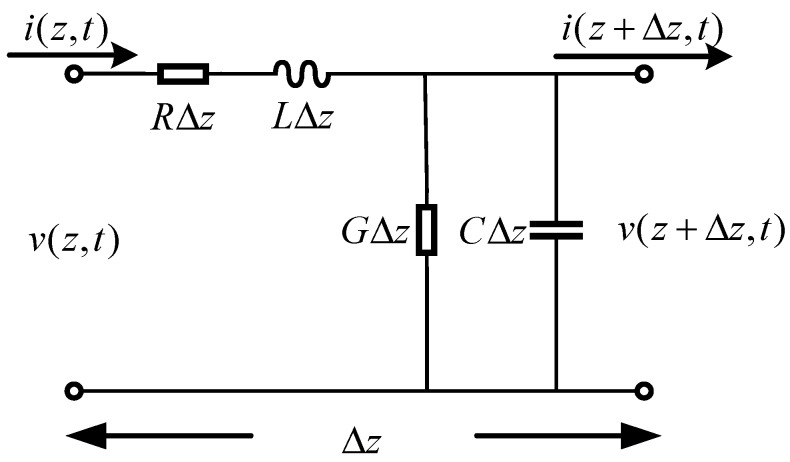
The transmission line model of the Bow-tie antenna.

Before the characteristic impedance and the shield impedance of the antenna are explained, the transmission line model of the Bow-tie antenna is supposed to be introduced based on the transmission line theory in Reference [21]. According to this theory, the antenna can be schematically represented as a two-wire line, since transmission lines always have at least two conductors. The piece of line of infinitesimal length Δ*z* of a transmission line can be modeled as a lumped-element circuit, as shown in Figure 2. Also, the antenna, a finite length of transmission line, can be viewed as a cascade of section of this lumped-element circuit. In the lumped-element circuit, R is series resistance per unit length, for both conductors, in ohm/m; L is series inductance per unit length, for both conductors, in H/m; *G* is shunt conductance per unit length, in S/m; *C* is shunt capacitance per unit length, in F/m. *R*, *L*, *G* and *C* are per unit length quantities and *L* represents the total self-inductance of the two conductors, *C* is due to the close proximity of the two conductors, *R* represents the resistance due to the finite conductivity of the conductors, *G* is due to dielectric loss in the material between the conductors. Therefore, *R* and *G* represent the loss. So, the characteristic impedance can be defined as below:

(2) $$Z_{k}=\sqrt{\frac{R+j\omega L}{G+j\omega C}}$$

However, as for a Bow-tie antenna, it is difficult to calculate its characteristic impedance by (2). In fact, its characteristic impedance is always calculated by [19]:

(3) $$Z_{k}=120\ln\cot\frac{\beta }{4}$$

where *β* is the cone angle of triangle fins.

**Figure 3:**
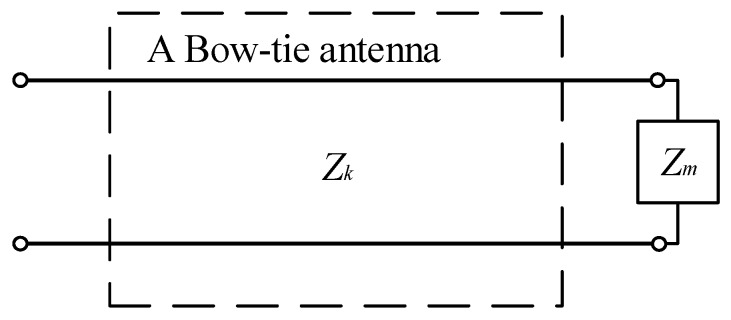
The Bow-tie antenna is terminated in a load impedance Z_m_.

In addition, based on transmission line theory, the antenna is regarded as a two-port network and is terminated in a load impedance *Z_m_*, which is shown in Figure 3. The load impedance illustrates wave reflection on antennas, and it can be defined as the ratio of voltage to current at the load. 

**Figure 4:**
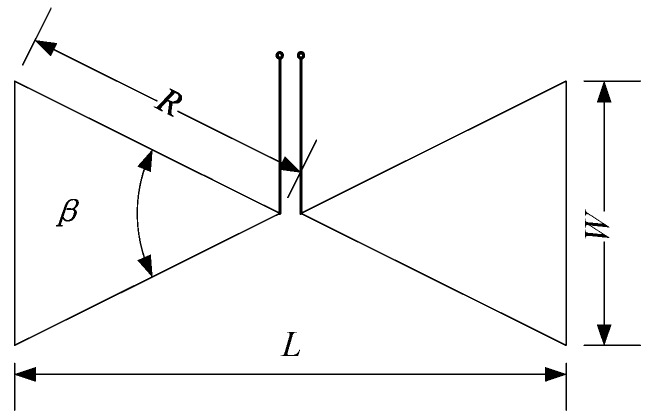
This is typical Bow-tie antenna geometry, the wideband of antenna depends on the cone angle β of triangle fins and the working frequency of antenna depends on its length *L*.

In addition, based on the Reference [22], the length of antenna can be calculated from characteristic impedance and working frequency by:

(4) $$L=\frac{\lambda }{2}(1-\frac{97.82}{Z_{k}})$$

where *λ* is the wavelength of the center frequency, L is the length of the antenna. Figure 4 shows the geometry of a typical Bow-tie antenna. According to (3) and (4), it can be concluded that on the one hand, the wideband of antenna depends on the cone angle *β* of triangle fins, on the other hand, the working frequency of antenna depends on its length *L*. 

However, the typical Bow-tie antenna sensor has some drawbacks for using in SFCW-GPR system. For example, its bandwidth is not wide enough and its radiation pattern is also not good enough. So, the typical Bow-tie antenna need to be modified. The main idea of modification is divided into 4 main steps:

- Step 1: two angles in every triangle fin of the Bow-tie antenna are removed, because the size of the antenna can be reduced without its electrical performance changed.
- Step 2: a slot is etched on each side of triangle fins, because it is used as capacitive load to improve the antenna input impedance;
- Step 3: there are two 50-ohm resistors placed at either terminal of each slot;
- Step 4: the cavity is loaded based on image theory.

**Figure 5:**
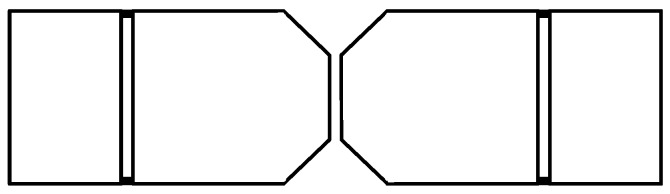
This is the geometric layout of the MOA. The antenna is printed on the FR-4 substrate, whose overall dimension is 177.75 × 91.5 × 2 mm^3^ with a thickness of 2.0 mm, loss tangent of 0.02 and fractional permittivity of 4.4.

Figure 5 shows the geometric layout of the MOA. The antenna is printed on the FR-4 substrate, whose overall dimension is 177.75 × 91.5 × 2 mm^3^ with a thickness of 2.0 mm, loss tangent of 0.02 and fractional permittivity of 4.4. The length of the patch L, which determines the minimum working frequency, is 132.5 mm. 

**Figure 6:**
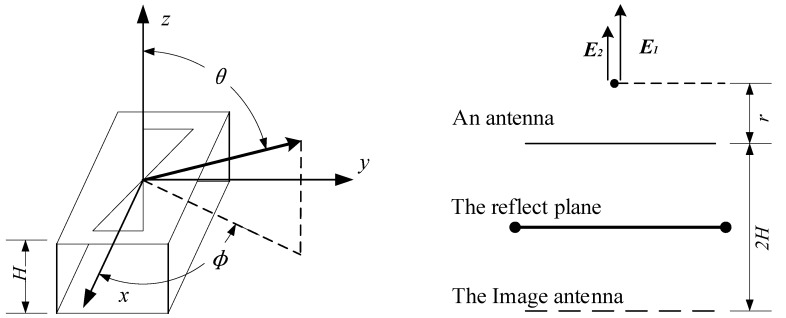
These two pictures show the principle of cavity. (**a**) A piece of Bow-tie copper sheet mounted on an infinite reflect plane is displayed; (**b**) The structure of reflect plane produces an image antenna to improve the directivity of antenna.

In addition, the width of the gap on each fin is 2.0 mm, which is designed for modifying the antenna impedance and loading resistance. The value of resistance is 50-ohm, which is confirmed by the conclusion presented by Wu and King [23], and the value of input impedance is also supposed to be taken 50-ohm. Also, based on Reference [20], in order to improve the directivity of antenna and electric field intensity to the ground, the height of its cavity H, which consists of copper sheet and four copper pillars, is supposed to be:

(5) $$H=\lambda_{0}/4$$

where *H* is the height of cavity and *λ*_0_ is the wavelength of the center frequency. This paper considers a piece of Bow-tie copper sheet mounted on an infinite reflect plane, as shown in Figure 6. The far-zone radiated field *E_θ_*, *E_φ_* can be written as:

(6) $$E_{\theta }=j\frac{abkE_{0}e^{-jkr}}{2\pi r}\{\sin\phi [\frac{\sin X}{X}][\frac{\sin Y}{Y}]\}$$

(7) $$E_{\phi }=j\frac{abkE_{0}e^{-jkr}}{2\pi r}\{\cos\theta \sin\phi [\frac{\sin X}{X}][\frac{\sin Y}{Y}]\}$$

where

(8) $$X=\frac{ka}{2}\sin\theta \cos\phi$$

(9) $$Y=\frac{kb}{2}\sin\theta \sin\phi$$

(10) $$k=\omega \sqrt{\mu \varepsilon }$$

When *θ = 0, φ = 0*, The far-zone radiated field *E_1_* and *E_2_* can be written as:

(11) $$E_{1}=E(\theta =0{}^{\circ},\phi =0{}^{\circ})=j\frac{abkE_{0}e^{-jkr}}{2\pi r}$$

(12) $$E_{2}=E(\theta =180{}^{\circ},\phi =0{}^{\circ})=-j\frac{abkE_{0}e^{-jk(r+2H)}}{2\pi (r+2H)}$$

where *a* is the length of Bow-tie antenna and *b* is the width of antenna. Finally, the overall far-zone radiated field E can be written as:

(13) $$E=E_{1}+E_{2}=\underset{r\to \infty }{\lim}[E_{1}(1-\frac{re^{-j2kH}}{r+2H})]=E_{1}(1-e^{-j2kH})$$

It must be noted that the dielectric between the antenna and the reflect plane is air, so *k = ω =* 2*π/λ_0_*, and E can be further rewritten as:

(14) $$E=E_{1}(1-e^{-j\frac{4\pi H}{\lambda_{0}}})$$

where *E* is the total field strength to the ground and *E*_1_ is the electric field strength generated by a single antenna.

**Figure 7:**
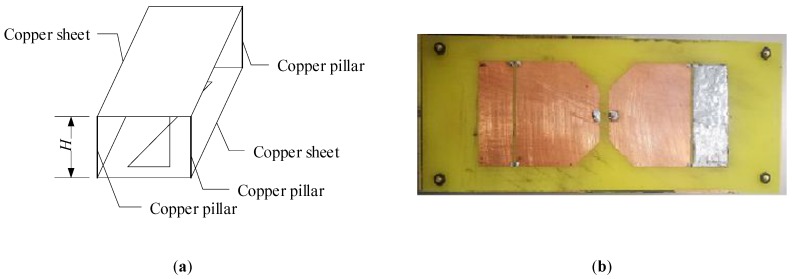
(**a**) This is the general view of modified antenna; the reflection cavity consists of a piece of copper sheet and four copper pillars; (**b**) This is the photo of antenna’s fin made by copper sheet.

At last, Figure 7 shows the general view of modified antenna and the fabricated Bow-tie antenna. The whole design parameters are presented in Table 3. Compared to other published bowtie structures, this structure is big because on the one hand, the reflector of antenna is flat sheet reflector, in order to make the image theory accounts adequately for the radiation pattern in direct and reflected fields, the reflector must be big enough; on the other hand, it is obvious that the electric size of antenna is positively correlated with the maximum wavelength of electromagnetic wave it receives. It means that the lowest frequency of this Bow-tie antenna is 700 MHz, which limits the miniaturization of antenna. In addition, differently with other Bow-tie structures, in the application of GPR system, the energy of electromagnetic wave the antenna receives is so small that the signal is difficult to be detected. So, according to [15,24,25], most of the structures of GPR antennas have been very big so far and the miniaturization of GPR antenna is still a difficulty.

### 2.2. The Modification and Improvement in Frequency Domain

Before the steps of modification and improvement, there are two important indexes to be introduced. First, voltage standing-wave ratio (VSWR) of the antenna varies with the working frequency in practice. In order to make the antenna work properly, the VSWR in designed frequency range is limited. The second one, reflection coefficient, describing the energy loss caused by reflected echoes in the process of transmitting electromagnetic signals, is usually used to characterize the antenna’s operation bandwidth. 

In this section, there are three steps about antenna design where the first one is modification, the second one is improvement and the third one is reflection cavity loading. Based on finite element method (FEM) simulation with HFSS, the simulation results of three steps are given below.

#### 2.2.1. The Simulation of the Modification

**Figure 8:**
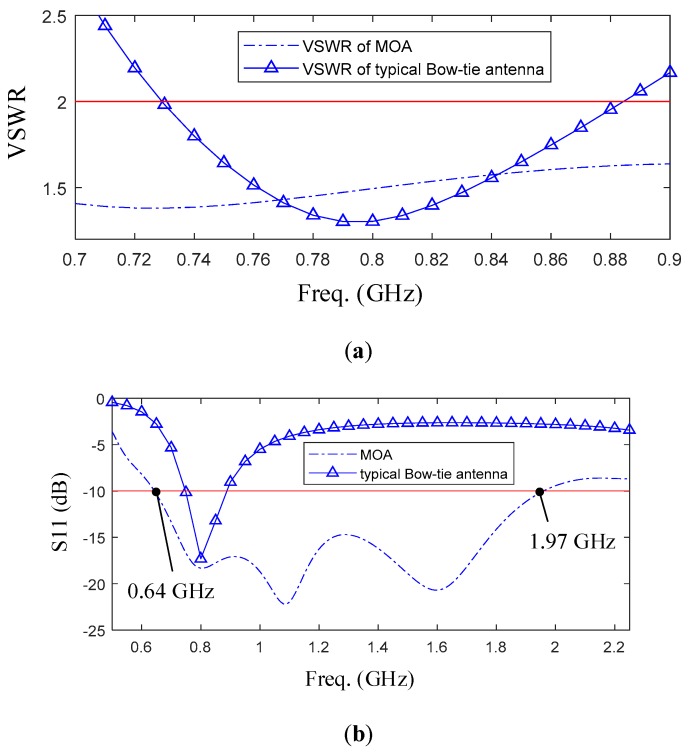
(**a**) Simulated VSWR for typical Bow-tie antenna and modified antenna; (**b**) The reflection coefficient of modified antenna.

In this modification, the VSWR of antenna is supposed to be less than 2.0. Figure 8a shows the simulated VSWR for typical Bow-tie antenna and modified antenna. the typical Bow-tie antenna has a bandwidth of 728 to 882 MHz under 2.0, and its fractional bandwidth is about 19%. By applying those modifications, the starting working frequency of antenna shift to a lower frequency of 0.64 GHz and the bandwidth has a wider range, which is from 0.64 to 1.97 GHz. What’s more, the fractional bandwidth of modified antenna become to 103.3%. Moreover, Figure 8b shows the relationship between the reflection coefficient and the frequency.

**Figure 9:**
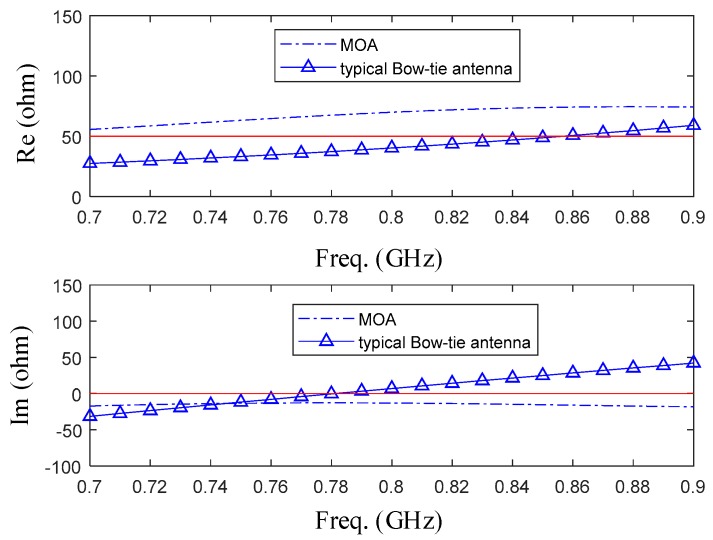
This picture shows the simulated impedance for typical Bow-tie antenna and modified antenna. Compared with the typical antenna, the imaginary part of input impedance of the MOA has been closer to 0.

The relationship between the frequency and input impedance of MOA and typical Bow-tie antenna is shown in Figure 9. Compared with the imaginary part of input impedance of typical antenna, the imaginary part of input impedance of the MOA has been closer to 0, but the distribution of the real part has become worse, which may affect the performance of antenna’s high frequency part.

**Figure 10:**
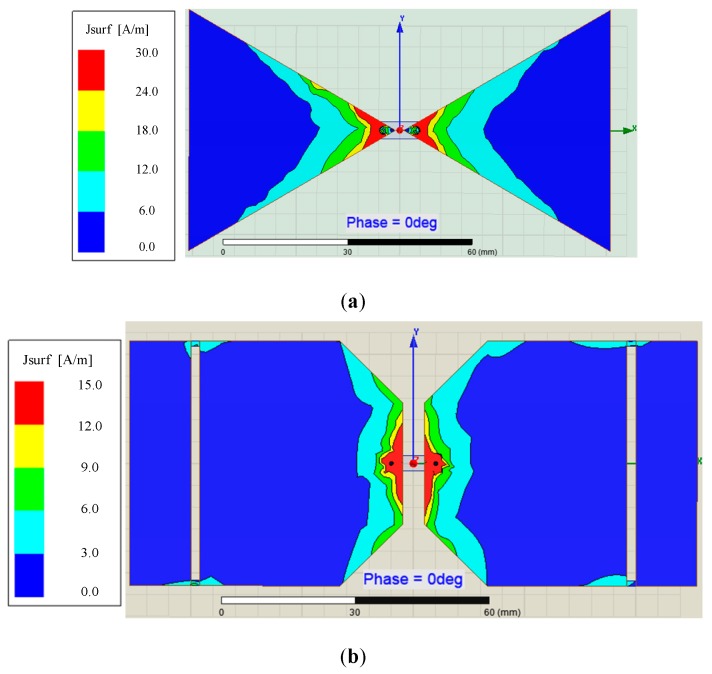

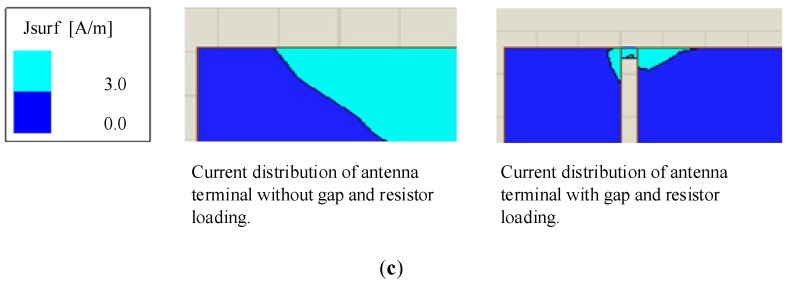
(**a**) Current distribution of the typical Bow-tie antenna; (**b**) Current distribution of the MOA; (**c**) Comparison of current distribution between two antennas.

As for current distribution, Figure 10a,b shows that of the typical Bow-tie antenna and the MOA separately, and Figure 10c shows the comparison of that at the end of fins between two antennas. 

Next, the simulation about the current distribution in different frequency has been done. Obviously, the current distribution on the typical Bow-tie antenna is worse than that on the MOA. First, the latter current distribution is more balanced than the former. Secondly, as far as the end of both antenna fins, the current tends to gather in the end of former fins rather than latter fins.

#### 2.2.2. Study and Simulation of the Improvement

**Figure 11:**
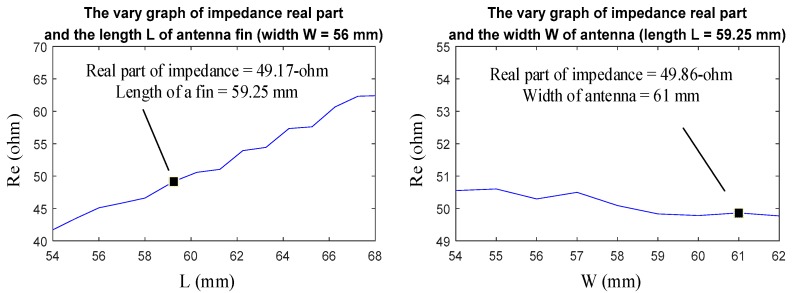
This picture shows the variation in center frequency of the real part of input impedance, the length of a fin *L* and the width *W*. From the label in the figures, it shows that the value of input impedance is the closest to 50-ohm when the *L* is 59.25 mm and *W* is 61 mm.

According to simulation results of Section 2.2.1., the performance of antenna in working frequency has been greatly modified, but in high frequency (HF) section the antenna didn’t perform very well. So, the length and width of antenna should be fine-tuned in order to improve the HF performance. Figure 11 shows the variation in center frequency of the real part of input impedance, the length of a fin *L* and the width *W*. It can be found out that the value of input impedance is the closest to 50-ohm when the *L* is 59.25 mm and *W* is 61 mm.

**Figure 12:**
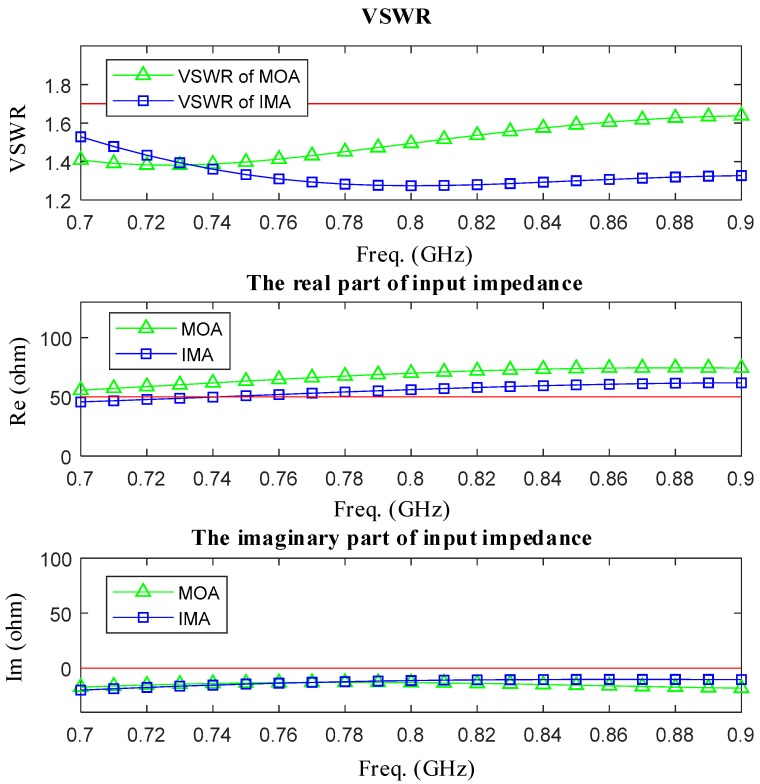
The simulated VSWR and input impedance of MOA and IMA are shown upon.

Then, Figure 12 shows the simulated VSWR and input impedance of MOA and IMA. Compared with the MOA, there are several obvious improvements on the new antenna. First, the resonant frequency has shifted from 720 MHz to 800 MHz and the distribution of VSWR has been much more uniform. Second, the real part of input impedance among the range of full working frequency has been closer to 50-ohm. Third, the imaginary part of input impedance among the range of full working frequency has been closer to 0.

**Figure 13:**
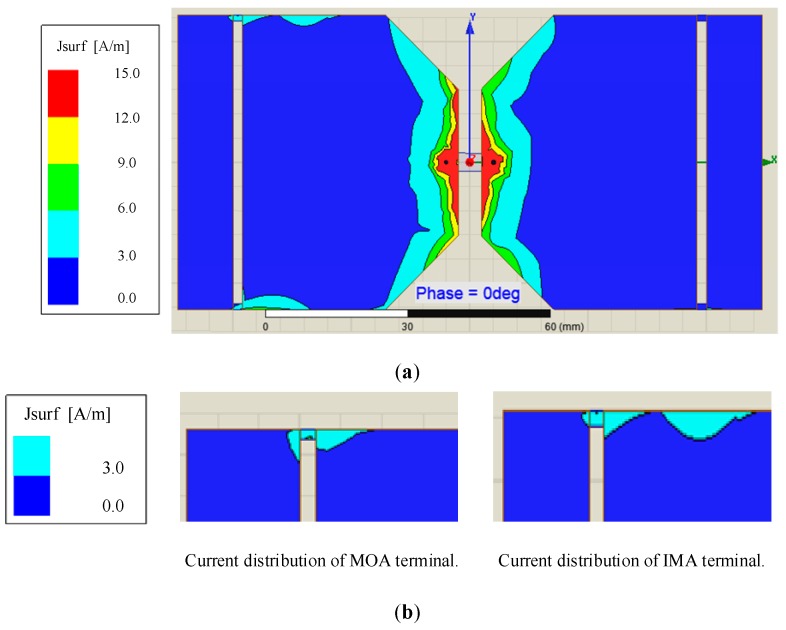
(**a**) Current distribution of the IMA; (**b**) Comparison of current distribution between two antennas.

Finally, the current distribution of the new antenna has also been improved. Figure 13a show the current distribution of IMA, and Figure 13b shows the comparison of that at the end of fins between the MOA and IMA. The simulation results show that the current aggregation around the resistance disappear after improved and the current aggregation effect of the end of antenna’s fins is further cut off.

#### 2.2.3. Study and Simulation of the Reflection Cavity

**Figure 14:**
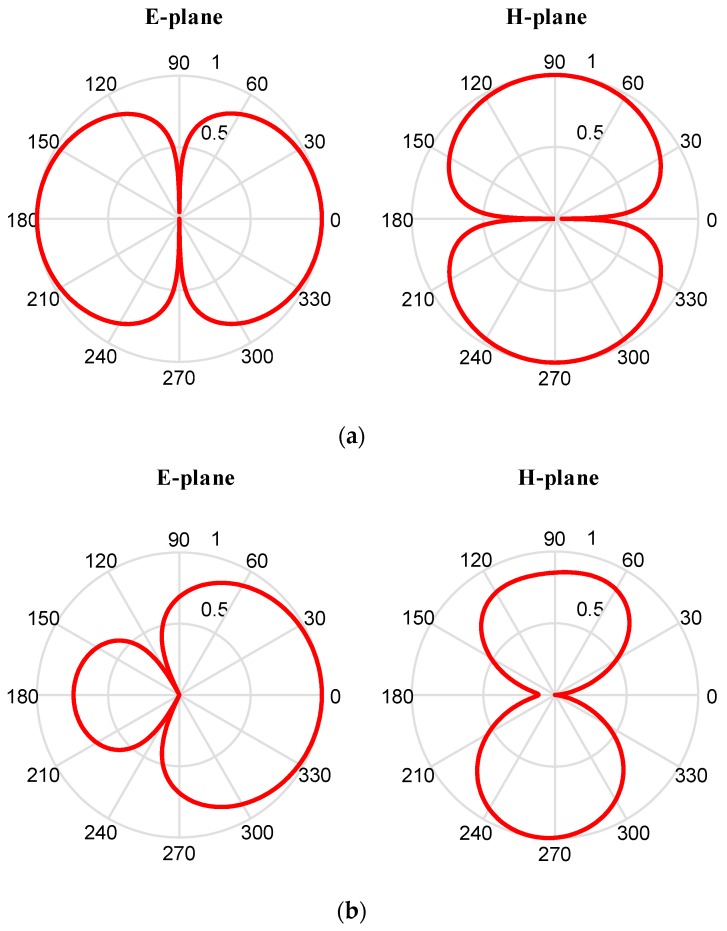
Comparison of radiation characteristic of two antennas: (**a**) The simulated E plane, H plane and 3D radiation pattern belong to the antenna without reflection cavity construction; (**b**) The simulated E plane, H plane and 3D radiation pattern belong to the antenna with cavity construction.

The simulated radiation patterns at 800 MHz of antennas with and without reflection cavity are shown in Figure 14. The E plane, H plane and 3D radiation pattern on the left belong to the antenna without reflection cavity construction, and that three patterns on the right belong to the antenna with cavity construction. For the E plane, the direction that 0-degree points to is where the ground is and it can be found out that the antenna directivity has been effectively improved by the reflection cavity structure. The 3D radiation patterns in the two pictures below also show that far field radiation of the backside of antenna has been obviously damped.

It is obvious that reflection cavity construction plays an important role in modifying the antenna’s radiation patterns. The gain pointing to −180 degrees is much lower than that of antenna without reflection cavity construction. The result shown in Figure 14 means that there is more energy radiated to ground by reflection cavity construction.

## 3. Results

### 3.1. The Measurement Results of Electrical Performance

**Figure 15:**
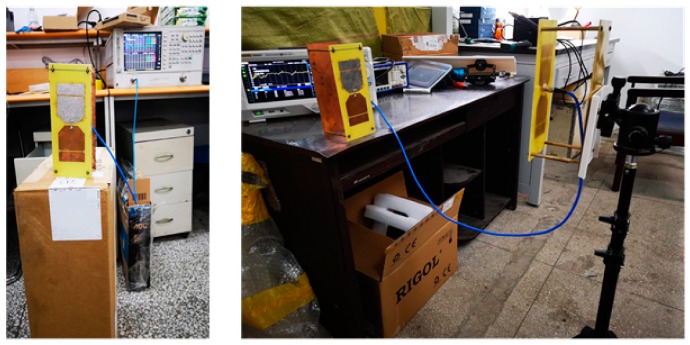
Two photos display the environment of measurement.

The performance of the antenna proposed prototype has been simulated and adjusted by using the HFSS (High Frequency Structure Simulator, Ansys Inc., south of Pittsburgh in Canonsburg, PA, USA). The performances of the antenna fabricated, including reflection coefficient, gain and input impedance, are measured by a PNA series vector network analyzer (HP8720ET 50 MHz–20 GHz, Agilent Technologies Inc., Santa Clara, CA, USA) and the step is shown in Figure 15. The data are analyzed and plotted by MATLAB (Matrix Laboratory, The MathWorks Inc., Natick, MA, USA).

**Figure 16:**
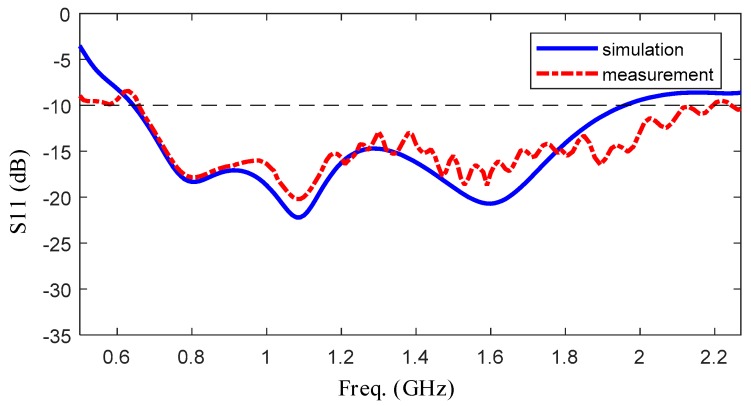
The comparison of reflection coefficient between measurement and simulation.

**Figure 17:**
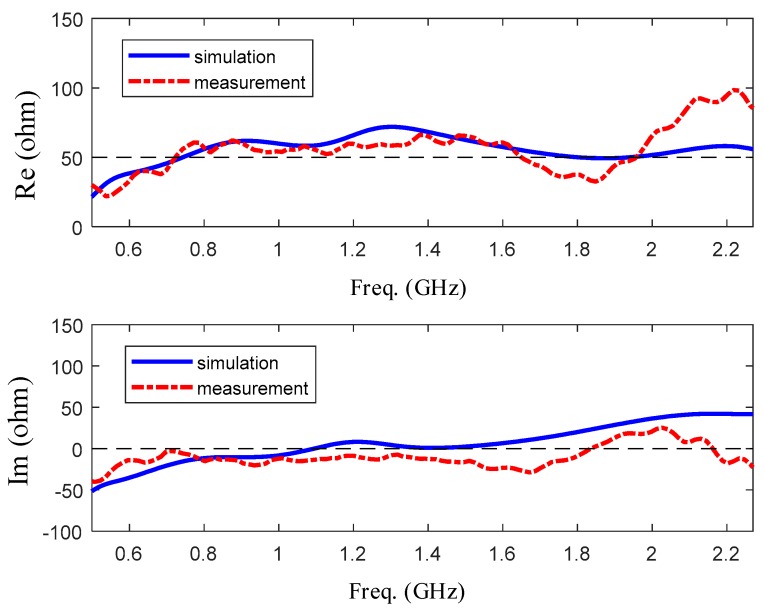
The comparison of input impedance between measurement and simulation.

The simulated and measured reflection coefficient and VSWR curves of the optimized design are plotted in Figure 16 and Figure 17. Figure 16 shows the comparison of reflection coefficient between measurement and simulation, and Figure 17 shows that of input impedance. As for reflection coefficient, it is shown that the fabricated antenna has an operation bandwidth of 1.6 GHz from 0.64 to 2.2 GHz. According to the measurement result, the highest resonant frequency is shown as 0.74 GHz. For input impedance, the measured real part of input impedance is around 50-ohm from 0.64 to 2.0 GHz and the imaginary part of that is around 0-ohm. To summarize, the fabricated antenna has a good frequency domain performance in the range of frequency from 0.64 to 2.0 GHz.

A modified gain measurement technique based on two-antenna method is used for this experiment. It basically refers to the Friis transmission equation in Reference [26], which is:

(15) $$\frac{P_{r}}{P_{t}}=\frac{A_{r}A_{t}}{{\left(r\lambda \right)}^{2}}$$

where *P_t_* is power fed into the transmitting antenna at its input terminals, *P_r_* is power available at the output terminals of the receiving antenna, *A_r_* is effective area of the receiving antenna, *A_t_* is effective area of the transmitting antenna, *r* is distance between antennas, *λ* is wavelength. Next, from Reference [19], assuming that the antenna is a lossless antenna, the gain of antenna can be represented as:

(16) $$G=\frac{4\pi A}{\lambda^{2}}$$

where *G* is the gain of antenna and *A* is effective area of the antenna. In addition, the *S*_21_ of a two-port network consisting of a transmitting antenna and a receiving antenna can be calculated by:

(17) $${\left|S_{21}\right|}^{2}=\frac{P_{r}}{P_{t}}$$

So, (15) can be expressed as:

(18) $${\left|S_{21}\right|}^{2}=G_{1}G_{2}{\left(\frac{\lambda }{4\pi r}\right)}^{2}$$

(19) $$\sqrt{G_{T}G_{R}}=\left|\frac{4\pi f}{c}\frac{\left|S_{21}\right|\left|S_{21}^{\prime }\right|}{\left|S_{21}\right|-\left|S_{21}^{\prime }\right|}\Delta r\right|$$

where *G_T_* is the gain of transmitting antenna and *G_R_* is the gain of receiving antenna. The gain of antenna can be calculated by (19) and the whole process of producing is summarized in [27] and the gain measurement technique is summarized in [28]. As is described in References [27,28] and Equation (19), Two identical fabricated antennas are needed in this method and two sets of transmission coefficient (*S*_21_ and *S*′_21_) in different distance between two antennas are supposed to be measured. In addition, the differences between two distances (Δ*r*) should also be recorded.

**Figure 18:**
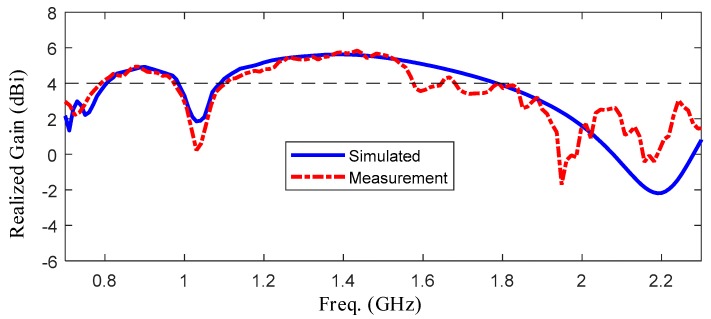
The comparison of realized gain between measurement and simulation.

**Figure 19:**
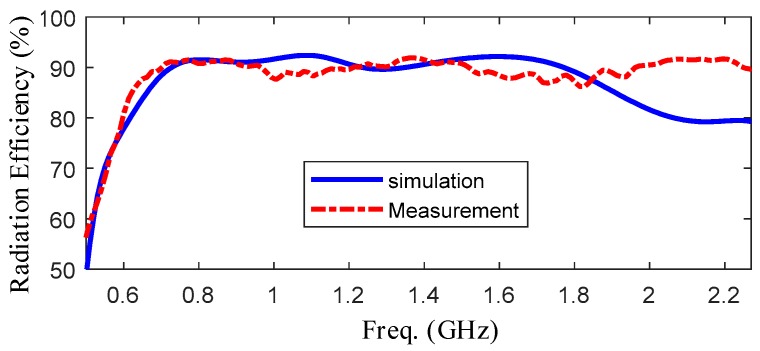
Radiation efficiency over working frequency.

Figure 18 shows the relationship between the gain and frequency. In this picture, blue line shows the simulated results and red line shows the measured results. It is obvious that the gain is fluctuating around 4 dBi and the peak of gain is higher than 5 dBi. Figure 19 shows the measured and simulated radiation efficiency of the UWB antenna with respect to frequency. From the picture, it can be seen that average radiation efficiency is around 90% with a maximum of 99%. From the experiments upon, a good agreement between the simulated and measured results was observed.

**Figure 20:**
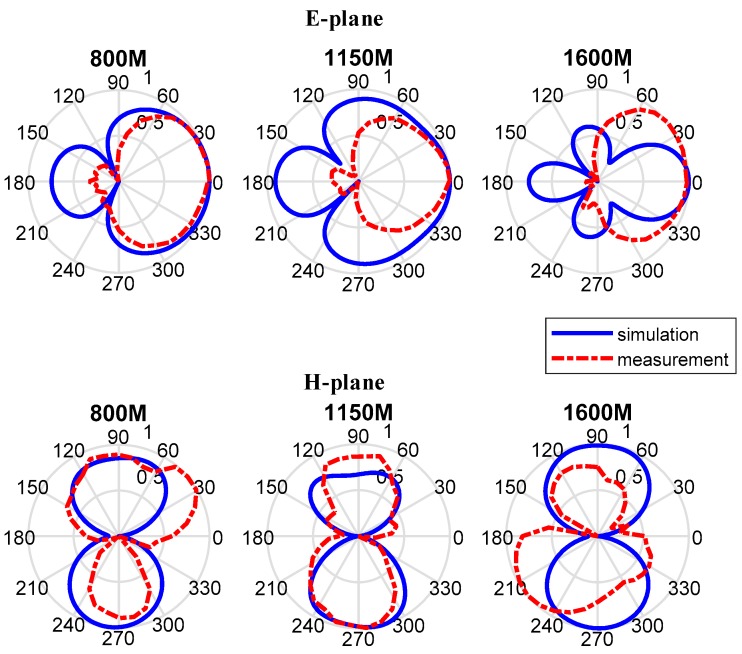
The measured and simulated results of E-plane (upon) and H-plane (below) and it can be found out that the measured back-lobe level at 1600 M at E-plane is considerably reduced as compared to 1150 M and 800 M.

In addition, the measured and simulated 2-D and 3-D radiation patterns are illustrated in graphs and pictures below for three different resonance frequencies of 800 MHz, 1150 MHz, and 1600 MHz. In the results, the *φ* and *θ* spherical coordinates are related to the Cartesian axis’s configurations. For example, *θ* = −180 degrees to 180 degrees and *φ* = 0 degree is the XZ-plane, *θ* = −180 degrees to 180 degrees, and *φ* = 90 degrees is the YZ-plane and *φ* = 0 degree to 360 degrees is the XY-plane whereas *φ* = 90 degrees. The XZ-plane (*φ* = 0 degree) is considered as E-plane and XY-plane (*θ* = 90 degrees) is considered as H-plane. 2-D patterns are shown in Figure 20 where red lines are measured results and blue lines are simulated results. From the far field measurement, the fabricated UWB antenna is directional and the main radiation direction is towards the ground and the key lobes of the radiation patterns are entirely towards the ground over the whole frequency band. The stable radiation pattern of the UWB antenna guarantees that the scattered signals have low noise density at the backward direction.

**Figure 21:**
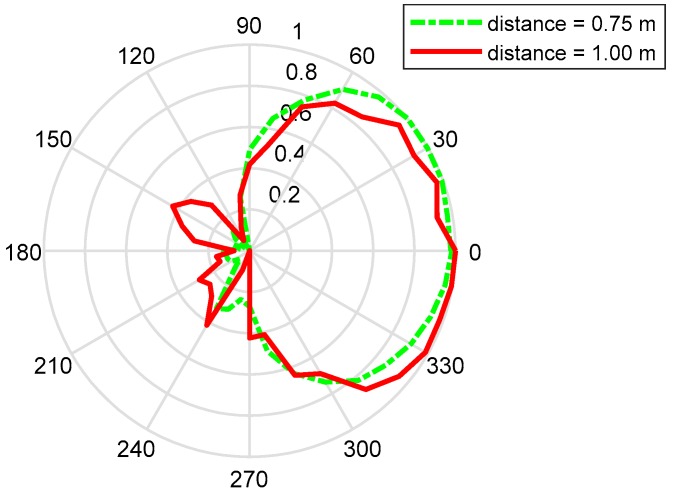
The polar pattern shows the comparison of radiation between the distance = 0.75 m and distance = 1.00 m. It is found out that the back-lobe level is different with the distance between the transmission antenna and receive antenna, and the back-lobe level of distance = 1.00 m is higher than that of distance = 0.75 m.

However, the measured back-lobe level at 1600 M at E-plane is considerably reduced as compared to 1150 M and 800 M. One possible reason is explained below. In GPR system, the antennas work in Fresnel region or radiation near field. In this field, the shape of the radiation pattern depends on the distance, in general. In addition, the measured results in Figure 20 is the results in radiation near field, and the range of radiation near field depends on the frequency. Although the distance between the transmission antenna and receive antenna is unchanged, the place where the receive antenna is in radiation near field is different with the frequency. So, this is the possible reason that the measured back-lobe level at 1600 M at E-plane is considerably reduced as compared to 1150 M and 800 M. Besides, another measurement is taken, and the results are shown in Figure 21. It is found out that the back-lobe level is different with the distance between the transmission antenna and receive antenna, and the back-lobe level of distance = 1.00 m is higher than that of distance = 0.75 m.

### 3.2. Dimensional Microwave Imaging Results and Discussions

**Figure 22:**
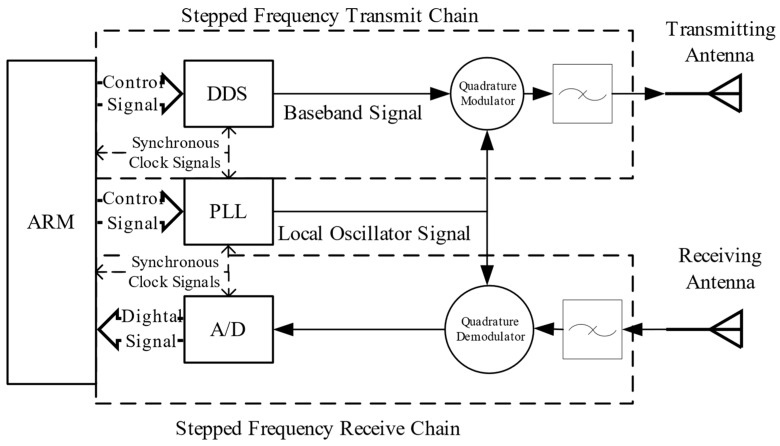
The structure of the experiment platform. In this figure, DDS is Direct Digital Synthesizer Unit, PLL is Phase Locked Loop Unit, A/D is Analog-to-Digital Converter and ARM is Advanced Reduced Instruction Set Computer Machines.

The main aim of this A-Scan measurement is to confirm that fabricated antennas can be used in SFCW-GPR system. An experimental platform is developed where the fabricated antennas is working as a transceiver, the signals are transmitted by a stepped frequency transmit chain and received by a receive chain. The stepped frequency transmit chain consists of four main parts including a Microcontroller Unit, a Direct Digital Synthesizer Unit (AD9959, Analog Devices, Inc., Norwood, MA, USA), a Quadrature Modulator Unit (AD8349, Analog Devices, Inc., Norwood, MA, USA) and a band pass filter. The receive chain consists of a band pass filter, a Quadrature Demodulator Unit (AD8347, Analog Devices, Inc., Norwood, MA, USA) and Analog-to-Digital Converter (AD9226, Analog Devices, Inc., Norwood, MA, USA). In addition, a Phase Locked Loop Unit (HMC830, Analog Devices, Inc., Norwood, MA, USA) is used to realize Clock Synchronization. the structure of it is shown in Figure 22.

This platform can produce the SFCW transmitted single from 0.7 to 0.9 GHz. The signal can be described by:

(20) $$x(t)=\sum_{n=0}^{N-1}\sin(2\pi (f_{0}+n\Delta f)t)rect(\frac{t-nT-T/2}{T})$$

(21) $$rect(t)=\{\begin{array}{cc} 1 & 0\leq t\leq 1\\ 0 & other \end{array}$$

where *x(t)* is the transmitted signal, *N* is the number of stepped frequency pulse, *f_0_* is the starting frequency, *Δf* is stepped frequency interval and *T* is pulse width. Table 4 lists the partial parameters.

**Figure 23:**
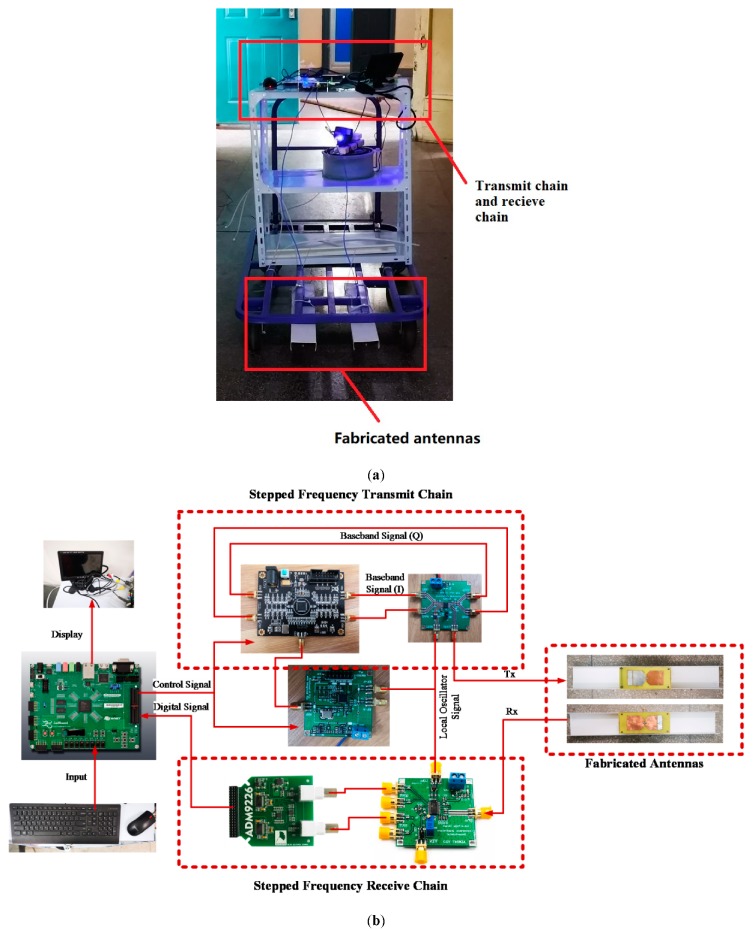
(**a**) The photo of experimental platform; (**b**) the transmit chain and receive chain is zoomed in this figure.

Otherwise, the system setup is illustrated in Figure 23 where two fabricated antennas are placed in the side by side X direction at a distance of 140 mm. In addition, the distance from the antenna to the ground is about 20 mm.

After receiving the frequency domain echoes, time domain echoes are recovered by Inverse Discrete Fourier Transform (IDFT) based on the frequency domain echoes. IDFT transformation process can be described by:

(22) $$x(n)=\frac{1}{N}\sum_{k=0}^{N-1}X(k)W_{N}^{-nk},(0\leq n\leq N-1)$$

(23) $$W_{N}=e^{-j\frac{2\pi }{N}}$$

where *x*(*n*) is restored time series signals, *N* is the length of time series signals, *X*(*k*) is a coefficient of *k*th harmonic sequence. 

**Figure 24:**
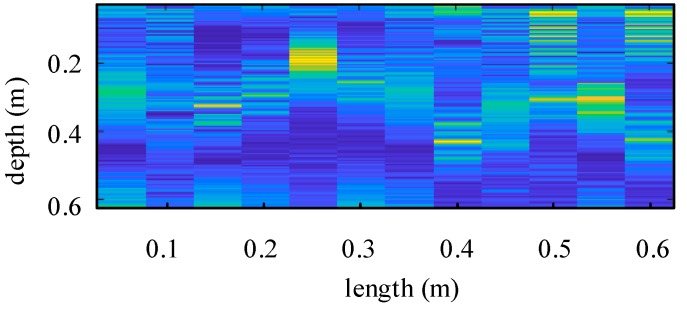
B-Scan echo in single channel.

The experiment is designed for detecting the reinforcement bar underground, and the measured echo is shown in Figure 24. The characteristic echo of reinforcement bar will be simulated in step 1 below and extracted from measured echo in step 2.

Then, the B-scan echo will be analyzed below, and the analysis is divided into two steps:

- Step 1: a simulation about the detection of multi-target underground is done, and the targets in this simulation is reinforce bar, so the simulated echo is the characteristic echo of reinforce bar. Pay attention to that the number of reinforce bar is a random setting. Also, the simulation will be repeated many times in order to obtain enough characteristic echoes and build a library of characteristic echoes of ideal reinforce bars.
- Step 2: Extract the feature lines from the B-Scan echo, and then match the feature lines with the characteristic echoes based on person correlation coefficient. If the correlation coefficient *r* is bigger than 0.8, the two groups of results will be related.

**Figure 25:**
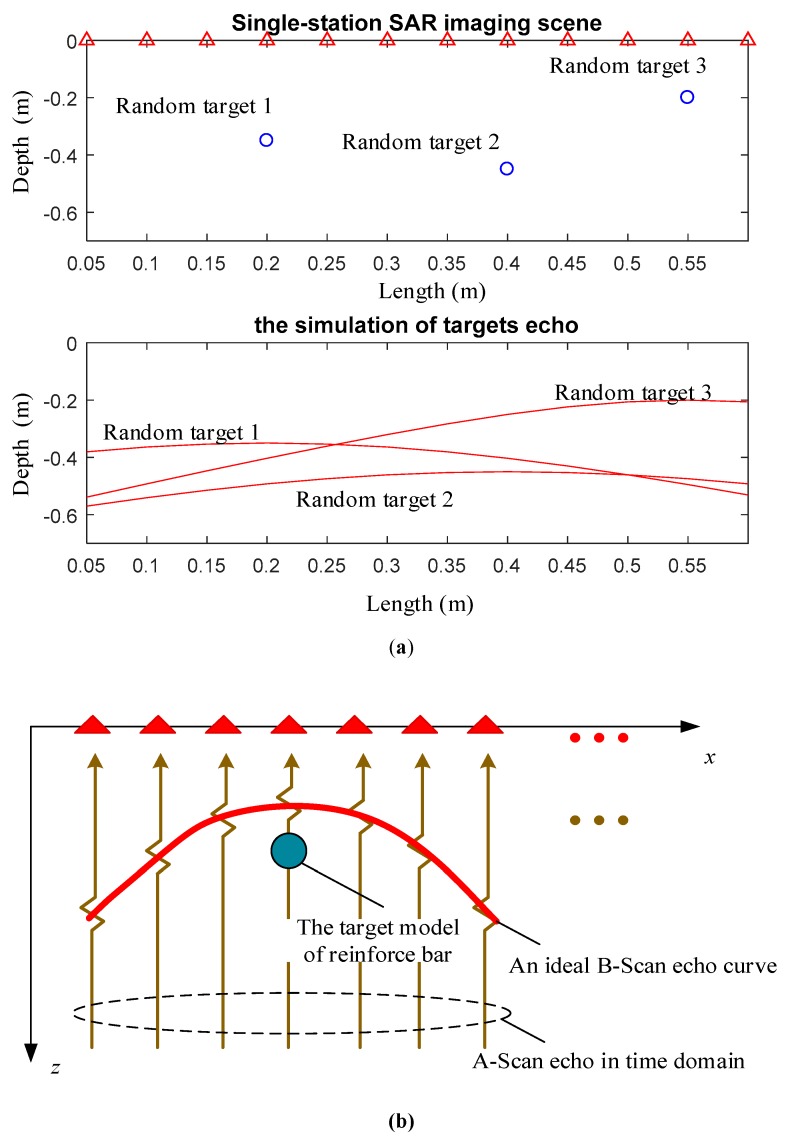
(**a**) This picture shows one random example described in step 1, where the section upon shows physical scene of Single-station Synthetic Aperture Radar (SAR) imaging and the section below shows the simulated B-scan echoes. Specifically, this is a random example from the library of characteristic echoes of ideal reinforce bars. (**b**) This picture shows how the upper figure derive the lower figure in (**a**), which is based on the multipath effect of electromagnetic wave.

In step 1, there is only one random example from a great number of simulations that this paper analyses. In this example, there are three reinforce bar to be detected. For the convenience of simulation, the physical model can be simplified to the structure displayed in Figure 22, where the triangle is T-R antennas and the three blue circles are idea reinforce bar. For example, as far as 3 targets measuring scene, antennas move from *X*1 to *X*12, there will be 12 A-Scan echoes (one-dimensional time-domain echo) collected. Then the B-Scan echo (two-dimensional time-domain echo image) can be obtained by the 12 A-Scan echoes. Time delay corresponding to each measuring point can be calculated by:

(24) $$\begin{array}{ccc} \tau_{n,k}=\frac{2\sqrt{{\left(x_{k}-x_{n}\right)}^{2}+z_{n}^{2}}}{c} & k=1,2,\ldots ,12 & n=1,2,3 \end{array}$$

where *τ_n,k_* is round trip time delay, *X_k_* is the place of T-R antennas, *X_n_* is the place of target n, *Z_n_* is the depth of target n. It is observed that the red curves in Figure 25a are hyperbolas and the coordinates of the targets can be obtained by calculating the focus of hyperbola. In addition, Figure 25b shows how the upper figure derive the lower figure in (a), which is based on the multipath effect of electromagnetic wave. That means, if *τ_n,k_* is measured, the distance between T-R antennas and targets will be calculated by (24). Also, Equation (24) is a mathematical expression of curve, and the place of the blue circle in the upper figure will be written as (*x_n_*, *z_n_*) if *τ_n,k_* is measured.

**Figure 26:**
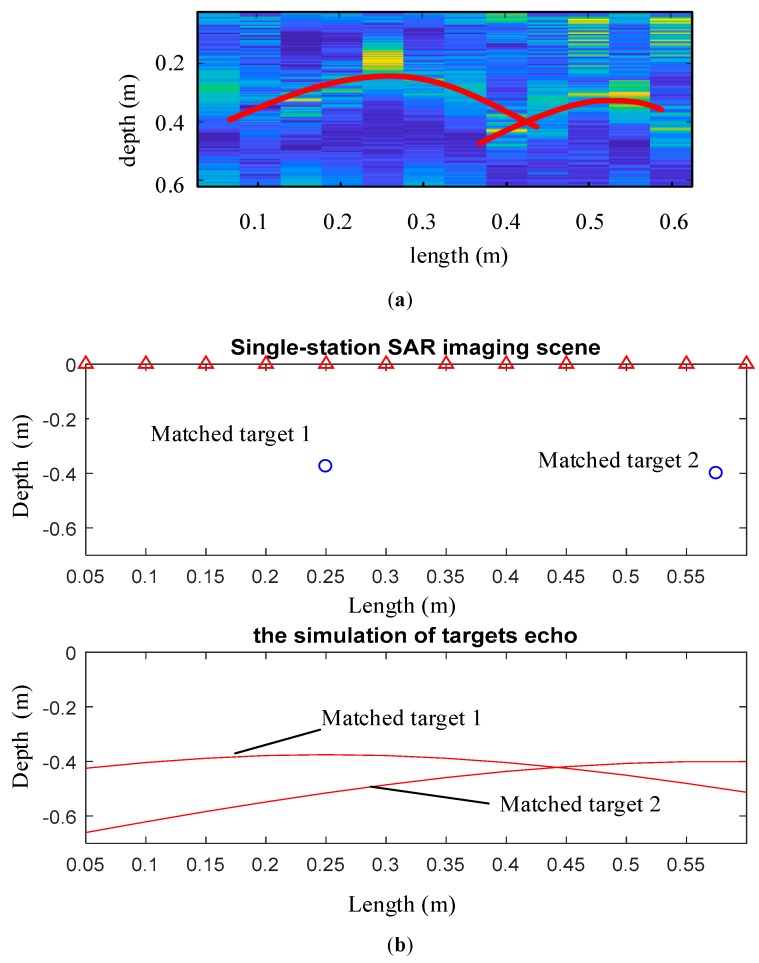
This picture shows the matching process described in step 2: (**a**) This section shows the characteristic echoes extracted from experiment results; (**b**) This section shows the matching result whose correlation coefficient *r* is 0.83.

In step 2, the feature lines from the B-Scan echo are extracted, and the feature lines with the characteristic echoes are matched based on person correlation coefficient. From the collected B-scan echo, there are two red target feature lines can be extracted based on this theory, which are signed by the red curves in Figure 26a. Then, match these two feature lines with the big characteristic echo library. The matching result, whose correlation coefficient *r* is 0.83, bigger than 0.8, is shown in Figure 26b. So, these two detected targets can both be defined as reinforce bars.

It clearly confirms that the fabricated antennas can be applied to the working scenarios of SFCW-GPR. In addition, the proposed antenna is compared with other antennas in Table 5. Comparing the proposed antenna with others, it is observed that the IMA can be an eligible imaging sensor equipped on GPR system with broad frequency of operation and high gain.

## 4. Conclusions

SFCW-GPR is widely used as a tool for nondestructive testing of shallow soil surface targets, and its function is mainly based on the theory of refraction, reflection and scattering of electromagnetic wave in the discontinuity of dielectric constant. So, as an important part of SFCW-GPR, an improved modified cavity-backed UWB antenna is designed and fabricated based on a typical Bow-tie antenna. After modification steps and improvement steps, including the methods of lumped loads, cavity-backed loading and structure loading, the performance of antenna has been much better compared with the typical Bow-tie antenna. The simulations and measurements show that the UWB antenna has a fractional bandwidth of 103.3% from 0.64 to 2.0 GHz with 50-ohm input impedance and directional radiation pattern. 

Moreover, the results of verification experiment of Step Frequency Continuous Wave (SFCW) show that the antenna can be applied to the working scenarios of SFCW-GPR.

## Figures and Tables

**Table 1:** The OSD/DARPA classification scheme for devices/signals based on bandwidth.

Band Type	Fractional Bandwidth (*B_F_*)
Narrowband (NB)	0.00 < *B_F_* < 0.01
Wideband (WB)	0.01 < *B_F_* < 0.25
Ultra-Wideband (UWB)	0.25 < *B_F_* < 2.00

**Table 2:** The simulated performances of typical Bow-tie antenna, MOA, and IMA.

Type of Antenna	Typical Bow-Tie Antenna (800 MHz)	MOA	IMA
Size of Copper Sheet (mm^2^)	102 × 57.735	127.5 × 56	123.5 × 62
Frequency (GHz)	0.75–0.89	0.64–1.96	0.64–2.03
Realized Gain (dBi)	−0.67 (0.8 GHz) −0.27 (1.15 GHz) 0.61 (1.60 GHz)	4.80 (0.8 GHz) 3.26 (1.15 GHz) 4.33 (1.60 GHz)	3.75 (0.8 GHz) 3.03 (1.15 GHz) 4.57 (1.60 GHz)
VSWR	1.34 (0.8 GHz) 4.79 (1.15 GHz) 6.66 (1.60 GHz)	1.36 (0.8 GHz) 1.31 (1.15 GHz) 1.14 (1.60 GHz)	1.28 (0.8 GHz) 1.25 (1.15 GHz) 1.20 (1.60 GHz)
Reflection Coefficient (dB)	−16.73 (0.8 GHz) −3.68 (1.15 GHz) −2.63 (1.60 GHz)	−16.19 (0.8 GHz) −17.56 (1.15 GHz) −23.53 (1.60 GHz)	−18.34 (0.8 GHz) −18.97 (1.15 GHz) −20.70 (1.60 GHz)
Radiation Efficiency (%)	95.14% (0.8 GHz) 54.19% (1.15 GHz) 42.56% (1.60 GHz)	93.53% (0.8 GHz) 93.25% (1.15 GHz) 94.56% (1.60 GHz)	92.59% (0.8 GHz) 93.73% (1.15 GHz) 94.15% (1.60 GHz)
Bandwidth (%)	17.07%	101.54%	104.12%
Type of Antenna	Bow-tie antenna	Bow-tie antenna	Bow-tie antenna

**Table 3:** Design Parameters of the MOA.

Parameter	Value
Material of substrate	FR-4
Relative permittivity of substrate	4.4
Substrate dimension	177.75 × 91.5 × 2 mm^3^
Height of cavity	74.25 mm
Diameter of copper pillar	3 mm
Length of antenna	132.5 mm
Width of antenna	56 mm
Width of gap	2 mm
Depth of each fin cut out	54.75 mm
Cone angle	90 degrees
Value of resistance	50-ohm
Number of resistances	4
Width of gap between two fins	5 mm

**Table 4:** Platform’s parameter setting.

Parameter	Value
the number of stepped frequency pulse (*N*)	34
starting frequency (*f*_0_)	700 MHz
ending frequency (*f_n_*)	904 MHz
stepped frequency interval (Δ*f*)	6 MHz
pulse width (*T*)	14 us

**Table 5:** Comparison between different antennas with the proposed IMA.

Ref. No.	[6]	[7]	[8]	This Work
Size (mm^2^)	36 × 36	130 × 130	180 × 300	177.75 × 91.5
Frequency (GHz)	2–6	2.1–3.1	0.26–0.4	0.64–2.0
Peak Gain (dBi)	7.5	5.0	- *	5.1
Bandwidth (%)	100%	39.22%	42.42%	103.3%
Type of Antenna	planar spiral antenna	Archimedean spiral antenna	half-ellipse antenna	Bow-tie Antenna

* Unable to find data.
